# Malignant Catarrhal Fever Induced by *Alcelaphine herpesvirus 1* Is Associated with Proliferation of CD8^+^ T Cells Supporting a Latent Infection

**DOI:** 10.1371/journal.pone.0001627

**Published:** 2008-02-20

**Authors:** Benjamin Dewals, Christel Boudry, Frédéric Farnir, Pierre-Vincent Drion, Alain Vanderplasschen

**Affiliations:** 1 Immunology-Vaccinology (B43b), Department of Infectious and Parasitic Diseases, Faculty of Veterinary Medicine, University of Liège, Liège, Belgium; 2 Biostatistics (B43), Department of Clinical Sciences, Faculty of Veterinary Medicine, University of Liège, Liège, Belgium; 3 Animal facility (B23), University of Liège, Liège, Belgium; Cambridge University, United Kingdom

## Abstract

*Alcelaphine herpesvirus 1* (AlHV-1), carried by wildebeest asymptomatically, causes malignant catarrhal fever (WD-MCF) when cross-species transmitted to a variety of susceptible species of the *Artiodactyla* order. Experimentally, WD-MCF can be induced in rabbits. The lesions observed are very similar to those described in natural host species. Here, we used the rabbit model and *in vivo* 5-Bromo-2′-Deoxyuridine (BrdU) incorporation to study WD-MCF pathogenesis. The results obtained can be summarized as follows. (i) AlHV-1 infection induces CD8^+^ T cell proliferation detectable as early as 15 days post-inoculation. (ii) While the viral load in peripheral blood mononuclear cells remains below the detection level during most of the incubation period, it increases drastically few days before death. At that time, at least 10% of CD8^+ ^cells carry the viral genome; while CD11b^+^, IgM^+^ and CD4^+^ cells do not. (iii) RT-PCR analyses of mononuclear cells isolated from the spleen and the popliteal lymph node of infected rabbits revealed no expression of ORF25 and ORF9, low or no expression of ORF50, and high or no expression of ORF73. Based on these data, we propose a new model for the pathogenesis of WD-MCF. This model relies on proliferation of infected CD8^+^ cells supporting a predominantly latent infection.

## Introduction

Malignant catarrhal fever (MCF) has been described as a fatal lymphoproliferative disease of a variety of species of the *Artiodactyla* order that includes cattle. The main causative agents of MCF are two closely-related gammaherpesviruses of the *rhadinovirus* genus, *ovine herpesvirus 2* (OvHV-2) and *alcelaphine herpesvirus 1* (AlHV-1). These viruses cause no apparent disease in their natural host species. Sheep are naturally infected by OvHV-2 which is responsible for the sheep-associated form of MCF when cross-species transmitted to susceptible hosts such as cattle. Wildebeest (*Connochaetes taurinus*) carry AlHV-1 responsible for the wildebeest-derived form of MCF (WD-MCF) [1]. In sub-Saharan Africa, cross-species transmission of AlHV-1 to susceptible host species occurs throughout wildebeest grazing areas and largely affects cattle. In addition, WD-MCF has also been reported throughout the world in zoological collections where mixed artiodactyl species including wildebeest are kept [2]. Experimentally, WD-MCF can be induced in rabbits [3]. The lesions observed are very similar to those described in natural host species.

WD-MCF is a disease described as a combination of lymphoproliferation and degenerative lesions caused by unknown mechanisms [1], [4], [5]. This assumption on the pathogenesis of WD-MCF relies on a very limited number of studies. The proliferation of lymphoid cells in WD-MCF lesions was inferred from macroscopic and microscopic observations. Macroscopic lesions of MCF are characterized by a severe hypertrophy of secondary lymphoid organs [1]. The main histopathological findings in WD-MCF lesions are necrotic vasculitis and infiltration of mononuclear cells surrounding arteries and veins in almost all organs. This cellular infiltrate is characterised by a high mitosis index [1]. Studies performed in cattle and rabbits have shown that T lymphocytes are the predominant cells associated with the vascular lesions [4], [6]. Whether these T lymphocytes are infected and/or proliferate is unknown. Extensive *ex vivo* cultures of lymphoid cells isolated from animals with WD-MCF led to the production of cytotoxic large-granular lymphocyte (LGL) cell lines expressing CD4 or CD8. LGL were shown to carry AlHV-1 genome and to induce WD-MCF when transferred to naive subjects [5]. Finally, studies based on immunofluorescence and *in situ* hybridization detected a very low level of infected cells in lesions (10^−6 ^and 10^−4^, respectively) [7], [8]. The phenotype of these rare infected cells was not established.

To reconcile all the observations described above, a model was proposed and commonly accepted for the pathogenesis of WD-MCF. According to this model, the rare cells that support AlHV-1 infection could induce proliferation and deregulation of non infected cells. The latter cells could then be responsible for some of the lesions observed through their cytotoxicity [7], [8]. The present study was devoted to the pathogenesis of WD-MCF using the rabbit model. Our goal was to investigate whether *in vivo* AlHV-1 infection is associated with lymphoproliferation, to identify the phenotype of potentially proliferating cells, and finally, to determine if proliferating cells are infected or not. Our results demonstrate that WD-MCF is associated with extensive proliferation of infected CD8^+^ T cells that support a predominantly latent infection. Based on these data, we propose a new model for the pathogenesis of WD-MCF.

## Methods

### Cell lines and virus strain

Embryonic bovine lung cells (EBL, German collection of micro-organisms and cell cultures DSMZ ACC192) were cultured in Dulbecco's modified Essential Medium (D-MEM, Invitrogen Corporation) containing 10 % foetal calf serum (FCS) (Bio Wittaker). The pathogenic AlHV-1 C500 strain isolated from an ox with MCF [9] was provided by Dr D. Haig (Moredun Research Institute, UK). The virus was maintained by limited passage (<5) in EBL cells before being inoculated to rabbits.

### Induction of WD-MCF in rabbits

Specific pathogen-free New-Zealand white rabbits were housed individually. Two groups, each comprising four rabbits were used. Animals of both groups were inoculated intravenously with 3×10^6^ AlHV-1 infected (containing 5.2×10^9^ viral genomic copies as determined by quantitative PCR, see below) or mock infected EBL cells, respectively. Infected cells for inoculation were harvested from cultures in which CPE reached 90 % or more. Rabbits were examined daily for clinical signs. According to bioethical rules, rabbits were euthanized when rectal temperature remained higher than 40°C for two consecutive days. The animal study performed had been accredited by the local ethics committee of the University of Liège (Belgium).

### Antibodies

Monoclonal antibodies (mAb) directed against rabbit CD11b (198), IgM (NRBM), CD5 (KEN-5), CD4 (KEN-4), and CD8 (12C.7) were used according to the manufacturer recommendations (Serotec Inc.). mAb 15-A (VMRD Inc) raised against AlHV-1 glycoprotein complex gp115 was also used in this study [10].

### Cell suspension preparation

Peripheral blood mononuclear cells (PBMC) were prepared as described elsewhere [11]. Single-cell suspensions were prepared from the spleen and the popliteal lymph node as follows. Immediately after euthanasia, the organs were removed, delicately chopped and passed through a stainless steel sieve. Red cells were then lysated as described earlier [12] . Cells were washed extensively with PBS before use.

### Indirect immunofluorescent staining

Immunofluorescent labeling was performed in PBS containing 10 % FCS. For cell phenotyping, living cells were incubated on ice for 30 min with the mAbs described above as the primary antibody. Samples were then washed with PBS and incubated on ice for 20 min with Alexa-Fluor 633 nm-conjugated goat-anti-mouse IgG (H+L) (Alexa 633-GAM, 5 µg/ml; Invitrogen) as the secondary antibody. After washing, samples were eventually processed to detect 5-Bromo-2′-Deoxyuridine (BrdU) incorporated into cellular DNA (see below).

### *In vivo* incorporation of BrdU

**Figure 1:**
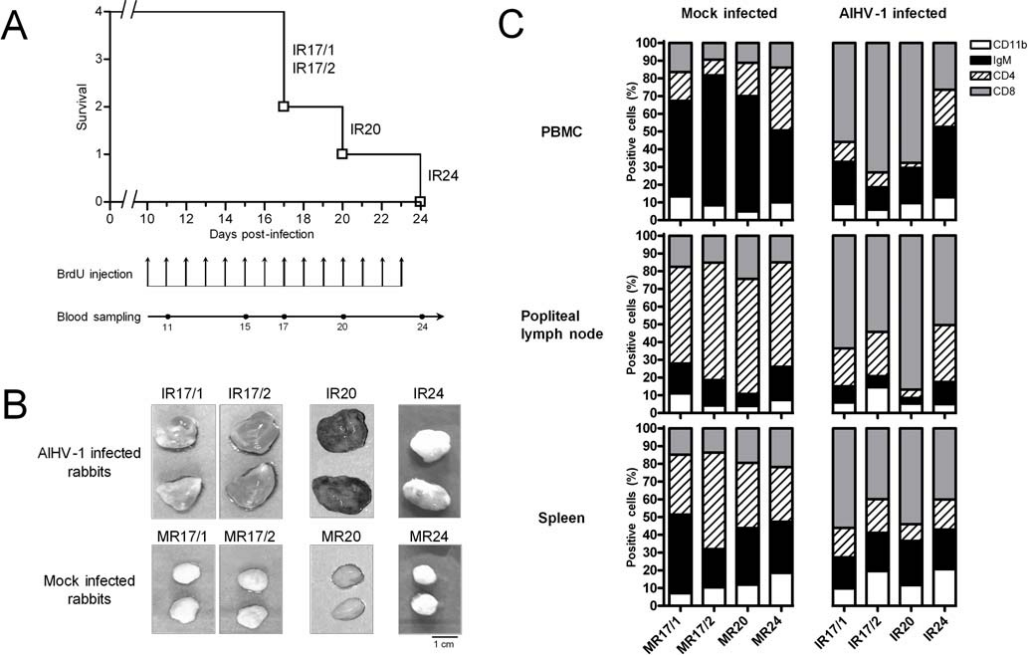
Induction of WB-MCF in rabbits. A. Cumulative incidence survival curve of AlHV-1 infected rabbits. On day 0, two groups, each consisting of four rabbits, were inoculated intravenously with 3×10^6^ mock infected or AlHV-1 infected EBL cells. At day 10 post-inoculation, BrdU (25 mg/kg) was injected intravenously every day to all rabbits. Rabbit developing WD-MCF were euthanized when rectal temperature remained higher than 40°C for two consecutive days. For each euthanized infected rabbit, a contemporary mock infected rabbit was also sacrificed. Rabbits were identified according to their infected (IR) or mock infected (MR) status and according to the time of death (see numbers). B. Popliteal lymph nodes were dissected during necropsy examination. C. Relative proportions of CD11b^+^, IgM^+^, CD4^+^ and CD8^+^ cells in PBMC, popliteal lymph node and spleen of AlHV-1 infected (IR) or mock infected rabbits (MR). At time of death, cell suspensions were prepared, stained and analysed by flow cytometry.

Starting on day ten post-AlHV-1 inoculation, rabbits were injected intravenously every day with BrdU (25 mg/kg; Sigma B5002) (Fig. 1). The number of cells going through the S phase *in vivo* was estimated by measuring the incorporation of BrdU by flow cytometry as described elsewhere with minor modifications [13], [14]. One million of immunophenotyped cells were washed with ice-cold PBS, and incubated overnight at 4°C in PBS containing 1 % (wt/vol) paraformaldehyde and 0.05 % (wt/vol) NP-40 (Fluka, Buchs, Switzerland). After being washed with PBS containing 1 % (wt/vol) glycine (Sigma-Aldrich), partial DNA denaturation was induced by incubation at 37°C for 30 min in PBS supplemented with 1 mg ml^−1^ of DNase I (Sigma DN-25; Sigma-Aldrich), 5×10^−3^ M CaCl_2_, and 10^−2^ M MgCl_2_. After an additional wash with PBS, the cells were resuspended in 20 µl of PBS containing 0.1 % (wt/vol) bovine serum albumin (Sigma-Aldrich) and 0.5 % (wt/vol) NP-40. BrdU staining was then performed by addition of 20 µl of anti-BrdU-FITC conjugated mAb (BD Biosciences) for 45 min at room temperature. Finally, cells were washed with PBS plus 0.1 % (wt/vol) bovine serum albumin prior to flow cytometry analysis. The threshold for BrdU positivity was set-up for each dot-plot manually.

### AlHV-1 quantitative PCR

DNA was extracted from cells using the QIAamp DNA mini kit (Qiagen) before analysis by quantitative PCR. The AlHV-1 ORF3 quantitative PCR was performed as described elsewhere [15], [16] (Table 1). An AlHV-1 BAC plasmid was used as a standard [12]. For cellular gene quantitative amplification, a 178 bp fragment of the rabbit β-globin was assayed as described elsewhere [15], [17] (Table 1). A 375 bp fragment of the rabbit β-globin gene (nt 337–711) was used as a standard. This fragment was amplified by PCR using the forward primer GlobF and the reverse primer GlobR (Table 1) and cloned into pGEM-T Easy. This PCR was able to detect 10 genome copies per sample (data not shown).

**Table 1:** Primers used in this study for PCR and RT-PCR reactions.

	Sequence	Genbank accession no.	Primer	Orientation	Primer sequence	Amplicon (bp)
**PCR**	Viral sequences					
	Formylglycineamide ribotide amidotransferase (FGARAT, ORF3)	AF005370	ORF3F	Forward	5′-GGGCTAATTTGTGCAGTTTGTGA-3′	111 bp
			ORF3R	Reverse	5′-AGGTGTTTCTGAAAAGAGGGGAA-3′	
	R transactivator (RTA, ORF50)	AF005370	C500-1	Forward	5′-TACGGGAGCCCTGACATTTCATCTCTTTTG-3′ a	405 bp
			C500-2	Reverse	5′-ATAACTGGTTGATGTGGCAGATGCATCTAT-3′ a	
	Cellular sequence					
	β-globin	V00882	qGlobF	Forward	5′-GGTATCCTTTTTACAGCACAAC-3′	178 bp
			qGlobR	Reverse	5′-CAGGTCCCCAAAGGACTCG-3′	
	β-globin	V00882	GlobF	Forward	5′-GTGGAAGAAGTTGGTGGTGAG-3′ a	375 bp
			GlobR	Reverse	5′-GTTCTCAGGATCCACGTGCAGC-3′ a	
**RT-PCR**	Viral sequences					
	Putative Latency Nuclear Antigen (ORF73)	AF005370	ORF73F	Forward	5′-GGACTAGACCCTCTTTATGACCC-3′	244 bp
			ORF73R	Reverse	5′-GCAATGGGTTCCTATTTTGCTCG-3′	
	R transactivator (RTA, ORF50)	AF005370	C500-1	Forward	5′-TACGGGAGCCCTGACATTTCATCTCTTTTG-3′ a	405 bp
			C500-2	Reverse	5′-ATAACTGGTTGATGTGGCAGATGCATCTAT-3′ a	
	DNA polymerase (ORF9)	AF005370	ORF9F	Forward	5′-GGATAGAAATGATCACGGGG-3′	1467 bp
	Major capsid protein (ORF25)	AF005370	ORF25F	Forward	5′-GAATGCCCTCCGAACTCTATG-3′	1541 bp
			ORF25R	Reverse	5′-CATAGTTCTGGATGTGGTTGTC-3′	
	Cellular sequence					
	β-globin	V00882	GlobF	Forward	5′-GTGGAAGAAGTTGGTGGTGAG-3′ a	249 bp
			GlobR	Reverse	5′-GTTCTCAGGATCCACGTGCAGC-3′ a	

a primers used for both PCR and RT-PCR reactions.

### PCR on sorted cells

Cells were sorted by flow cytometry using an automatic cell deposit unit (ACDU) in dry 96-well plates and directly frozen at -80°C overnight. Cells were then thawed on ice and suspended in water. PCR were performed on the cell lysate to detect AlHV-1 genome targeting ORF50. The forward primer C500-1 and the reverse primer C500-2 (Table 1) described earlier were used [12], [18]. Rabbit genomic DNA was detected by targeting a 178-bp sequence of the β-globin gene (nt 372–549) using the forward primer qGlobF and the reverse primer qGlobR [15], [17] (Table 1).

### RNA extraction and RT-PCR

Total RNA was isolated from cells using the RNeasy Mini Kit (Qiagen) with on-column DNase I digestion. Reverse transcription and PCR reactions were performed using the SuperScript III One-step RT-PCR System with Platinum *Taq* DNA polymerase Kit (Invitrogen). Gene-specific primers (Table 1) were used to detect viral mRNA or cellular β-globin mRNA. The absence of DNA template was verified by replacing the RT/*Taq* mix by the *Taq* DNA polymerase only.

### Flow cytometry and cell sorting

Flow cytometry acquisitions and analyses were performed using a three-laser Becton Dickinson fluorescence-activated cell sorter (FACSAria).

### Statistical analyses

Modelling and statistical analyses were performed using the SAS software. Based on 5 time points (11, 15, 17, 20 and 24 days) and 2 conditions (mock and AlHV-1 infected), with 4 rabbits in each situation (time×condition), a complete block design ANOVA for repeated measurements was used. Since some observations could not be considered independent, successive measurements on the same animal were correlated by including a random individual effect. Interaction between time and condition (*i.e.* kinetic of the two populations) was tested and *t*-tests were used in post-hoc tests to compare least square means. These tests used the estimation of the error variance based on the whole data set. The results presented in this manuscript are representative of three independent experiments.

## RESULTS

### Malignant catarrhal fever induced by AlHV-1 in rabbits is associated with proliferation of CD8^+^ cells

The initial goal of the present study was to compare *in vivo* PBMC proliferation between mock infected and AlHV-1 infected rabbits. Cell proliferation was investigated by *in vivo* labeling of proliferating cells with BrdU. BrdU is an analogue of thymidine that is incorporated into nascent DNA (cells going through the S phase) [19]. Intravenous injection of BrdU leads to staining of dividing cells throughout the body independently of the tissue of the cell type [20]–[22]. Due to the short half-life of the molecule *in vivo* only cells dividing at the time of the injection or shortly after are stained [23]. To circumvent this problem, BrdU was injected every day to mock and AlHV-1 infected rabbits starting on day 10 post-inoculation (Fig. 1A).

All AlHV-1 infected animals developed MCF clinical signs (apathy, anorexia, adipsia, hyperthermia and severe hypertrophy of popliteal lymph nodes) at 18±3.56 days post-inoculation (Fig. 1A). After two consecutive days of hyperthermia (≥40°C), rabbits were euthanized. At necropsy, examination of the organs of infected rabbits revealed characteristic MCF lesions including severe splenomegaly (the size of the spleens of infected rabbits was at least twice the size of those of mock infected rabbits) and generalized lymph node hypertrophy. The latter was particularly marked for the popliteal lymph nodes (the popliteal lymph nodes of infected rabbits were at least twice the size of those of mock infected rabbits) (Fig. 1B). Congestion and enlargement of the liver with greyish punctiform areas surrounding lobules was also detected, as well as irregular foci of various sizes (1–5 mm diameter) present within the cortical areas of the kidneys. For each euthanized infected rabbit, a contemporary mock infected rabbit was also sacrificed. None of the mock infected rabbits showed signs of illness during the course of the experiment or exhibited lesions at necropsy. These results suggest that the BrdU regime administrated to rabbits is not toxic on this short period.

At time of death, the relative proportions of CD11b, IgM, CD4 and CD8 expressing cells were estimated by flow cytometry in PBMC, popliteal lymph node and spleen (Fig. 1C). The results demonstrated that independently of the cell origin, AlHV-1 infected rabbits exhibited an increase of CD8^+^ cells associated mainly with a decrease of CD4^+^ cells.

**Figure 2:**
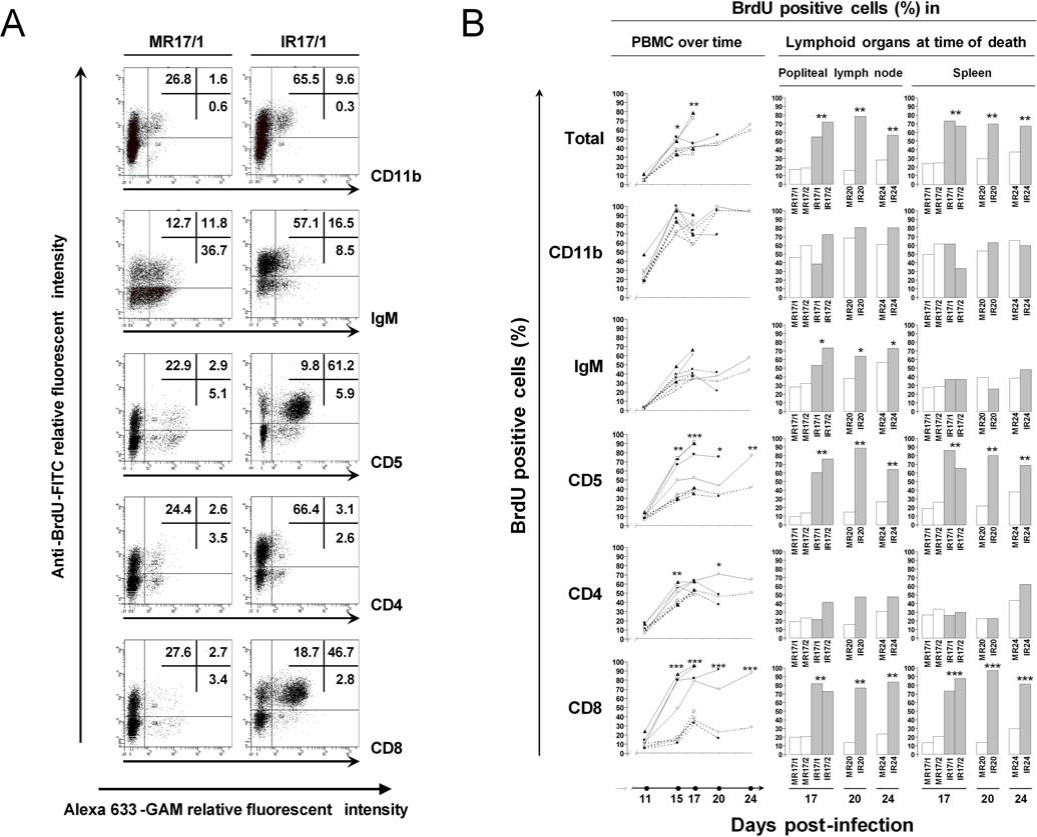
Analysis of *in vivo* BrdU incorporation. Rabbits were treated as described in Fig. 1. PBMCs were collected at days 11, 15, 17, 20 and 24 post-inoculation, while mononuclear cells were isolated from popliteal lymph node and spleen at the time of death. Cells were labelled with anti-CD11b, IgM, CD5, CD4 and CD8 mAbs as the primary antibodies. Alexa 633-GAM was used as the secondary antibody. *In vivo* BrdU incorporation was revealed by immunofluorescent staining as described in Methods. After staining, cells were analysed by flow cytometry. A. Representative flow cytometry dot plots are shown for each double staining, they illustrate the data obtained at day 17 post-infection for the PBMC of rabbits MR17/1 and IR17/1. The data represent the percentages of BrdU positive cells (*y*-axis) calculated based on the acquisition of 10,000 cells expressing the indicated cell marker (*x*-axis). B. The percentage of BrdU positive cells amongst the indicated cellular subset was determined and compared between AlHV-1 infected (left column: bold lines; middle and right columns: hatched bars) and mock infected (left column: dotted lines; middle and right columns: open bars) groups (* *P*<0.05; **P<0.005, *** *P*<0.0001). In the left column the following symbols were used: ▴, MR17/1 and IR17/1; □, MR17/2 and IR17/2; •, MR20 and IR20; ○, MR24 and IR24.

Cell proliferation was investigated in PBMC at different intervals after AlHV-1 inoculation and in secondary lymphoid organs at the time of necropsy (popliteal lymph node and spleen) (Fig. 2). BrdU incorporation and PBMC phenotypes were detected by double indirect immunofluorescent staining and flow cytometry analysis. Representative flow cytometry dot plots are presented in Fig. 2A. The specificity of the BrdU staining was demonstrated by treating cells from rabbit that were not injected with BrdU (data not shown). Statistical analyses of the data were performed with the SAS software in order to compare kinetics of BrdU labelling in the different cell populations analysed. Modelling was performed for each cell population (CD11b, IgM, CD5, CD4 and CD8) at the different time points of sampling (11, 15, 17, 20 and 24 days post-inoculation) and for the two groups of rabbits (mock infected and infected). Statistical significance for the kinetics was assessed through the interaction between time and treatment (mock infected and infected rabbits) (Fig. 2B). The results presented below are representative of three independent experiments.

As expected, the percentages of BrdU positive cells in mock infected and AlHV-1 infected groups increased gradually for all cell types over the time of the experiment. However, as early as 15 days post-inoculation, the percentage of BrdU positive cells was statistically higher in the infected group for some cell markers (CD4, CD5 and CD8). While for CD4+ cells the difference was statistically significant only on day 15 (*P*<0.005) and 20 (*P*<0.05), the difference was consistently observed for CD5 and CD8 positive cells and was highly significant for CD8^+^ cells (*P*<0.0005).

Finally, the SAS software was used to compare the kinetic of cell division observed for each cell subset between the mock infected and the infected groups. These analyses revealed no significant difference for CD11b^+^ (*P* = 0.2794), IgM^+^ (*P* = 0.2630) and CD4^+^ (*P* = 0.1365) cells. In contrast, a highly significant difference was observed for CD5^+^ (*P* = 0.0004) and CD8^+^(*P*<0.0001) cells.

The analyses performed on cells harvested from the popliteal lymph node and the spleen at necropsy led to similar conclusions. The percentages of BrdU positive cells amongst CD5^+^ and CD8^+^ cells were higher in the AlHV-1 infected group than in the mock infected one (*P*<0.005 or lower). In contrast to the observation made in PBMC, no significant difference in CD4 proliferation was observed in secondary lymphoid organs between the infected and the mock infected groups. Finally, a higher rate of proliferating IgM^+ ^cells was found in the lymph node (but not in the spleen) of infected rabbits compared to the mock infected group (*P*<0.05).

Altogether, the data presented above demonstrate that WD-MCF in rabbits is associated with abundant proliferation of CD8+ cells.

### AlHV-1 DNA load in PBMC increases drastically during the course of WD-MCF in rabbits

**Figure 3:**
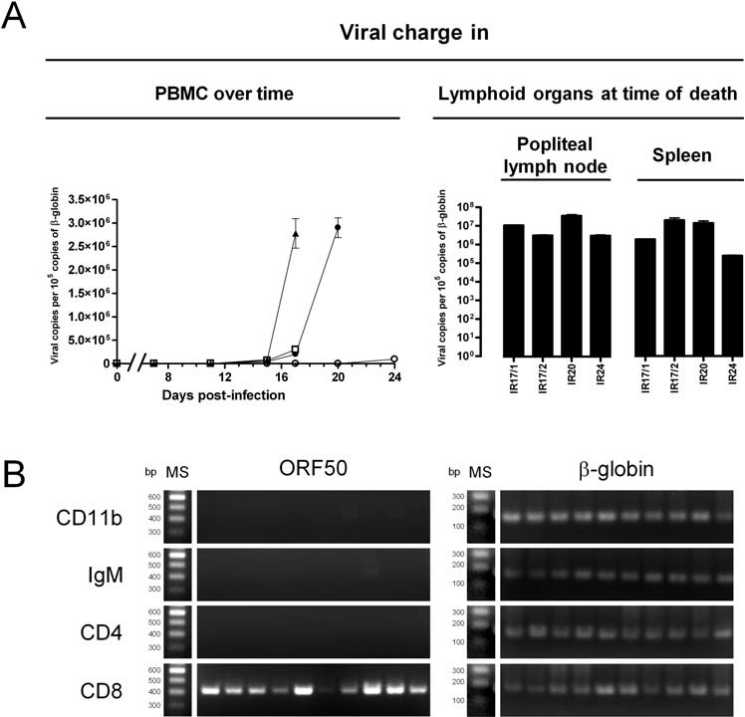
Load and tropism of AlHV-1 in PBMC and secondary lymphoid organs. Rabbits were treated as described in Fig. 1. A. Viral load in PBMC over time and in lympoid organs at the time of death. PBMC were collected at days 0, 7, 11, 15, 17, 20 and 24 post-infection, while mononuclear cells were isolated from popliteal lymph node and spleen at the time of death. DNA was extracted from cells and was subjected to viral (ORF3) and cellular (β-globin) real-time PCR quantification. The results are expressed as the estimated AlHV-1 mean genome copy number per 10^5^ cellular β-globin genomic copies. Data are the mean±SEM of triplicate measures. In the left panel, the following symbol are used: ▴, IR17/1; □, IR17/2; •, IR20; ○, IR24. B. AlHV-1 tropism amongst PBMC collected before euthanasia. Isolated cells were labelled with anti-CD11b, IgM, CD5, CD4 and CD8 mAbs as the primary antibodies, and Alexa 633-GAM, as the secondary antibody. For each mAbs, ten positive cells were sorted per well in 10 wells of a 96-well plate using an automatic cell deposit unit as described in Methods. After freezing at −80°C, PCR were performed in order to detect the AlHV-1 ORF50 and the cellular β-globin gene. Marker sizes (MS) are indicated on the left. The results presented in this figure were obtained with the PBMC of IR20. They are representative of the results obtained with the 4 infected rabbits.

To estimate the mean viral load in PBMC during the course of the disease, quantitative PCR assay was applied on samples collected at regular intervals (Fig. 3A, left panel). AlHV-1 genome mean copy number was normalized per 10^5^ copies of rabbit β-globin gene. Interestingly, the data presented in Fig. 3A (left panel) demonstrate that AlHV-1 viral load increased drastically only few days before death. This increase was particularly marked for rabbits IR17/1 and IR20. While their viral charge was still below the detection level on day 15 post-inoculation, it reached values of 2.78×10^6 ^and 2.90×10^6^ AlHV-1 copies per 10^5^ copies of rabbit β-globin 2 and 5 days later, respectively. Quantification of AlHV-1 genome copy in mononuclear blood cells isolated from lymphoid organs at the time of death revealed viral charge varying from 2.42×10^5^ to 37.86×10^6^ AlHV-1 copies per 10^5^ copies of rabbit β-globin (Fig. 3A, right panel). No correlation was observed between the viral charges measured in the different tissues.

### The tropism of AlHV-1 amongst PBMC is restricted to CD8^+^ cells

In order to determine which cell subset(s) amongst PBMC is (are) supporting AlHV-1 infection, PBMCs were collected from infected rabbits just before euthanasia. After staining, CD11b, IgM, CD4 and CD8 positive cells were sorted by flow cytometry using an ACDU system. 1, 5 or 10 cells were sorted per well. Sorted cells were then submitted to PCR to amplify rabbit β-globin gene and AlHV-1 ORF50. PCR performed on single sorted cell and 5 sorted cells led to inconsistent amplification of the β-globin gene (data not shown); most probably revealing cell and/or DNA lost during the procedure. In contrast, PCR performed on 10 sorted cells led to consistent amplification of the cellular gene (Fig. 3B). Consequently, the later number of sorted cells was used in this experiment. AlHV-1 ORF50 was amplified consistently from CD8 positive sorted PBMC (Fig. 3B). This result was observed with the four infected rabbits and was also obtained when analysing cells harvested from popliteal lymph node and spleen (data not shown). In contrast PCR reactions performed on CD11b, IgM and CD4 expressing cells did not generate ORF50 amplicon. A faint band was only observed in one out of 10 samples of CD11b (Fig. 3B, lane 7) and IgM (Fig. 3B, lane 9) sorted PBMC of rabbit IR20. Altogether, the results presented above suggest that the main tropism of AlHV-1 in rabbits developing MCF is CD8 positive cells. However, the data of the present study can not exclude the possibility that other cell types carry the viral genome with a lower frequency of infection than CD8 positive cells.

### AlHV-1 infected mononuclear blood cells support a predominantly latent infection

**Figure 4:**
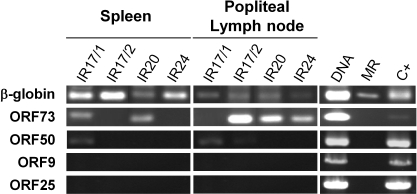
AlHV-1 gene expression in secondary lymphoid organs of rabbit with WD-MCF. Mononuclear cells were isolated from the spleen and the popliteal lymph node of rabbits at the time of death. Total RNA was extracted from isolated cells. Expression of cellular β-globin gene and viral genes (ORF73, ORF50, ORF9 and ORF25) was studied using the RT-PCR approach described in the Methods. DNA, purified AlHV-1 genomic DNA; MR, RNA extracted from the lymph node of a mock infected rabbit, C*+*, RNA purified from infected EBL cells.

The detection of AlHV-1 genome in mononuclear blood cells could reflect a permissive infection. In order to address this hypothesis, mononuclear blood cells were harvested from the popliteal lymph node and the spleen of infected rabbits. Indirect immunofluorescent staining of fixed and permeabilized cells using mAb 15-A (raised against AlHV-1 glycoprotein complex gp115) did not reveal the expression of its structural antigen (data not shown). Similarly, meticulous electron microscopic examination of the cells did not revealed indication of viral replication (data not shown). These results suggested that infected cells do not support a permissive infection. To test this hypothesis, we investigated the expression of specific AlHV-1 genes known to be transcribed during replication cycle and/or latency (Fig. 4). ORF9 encoding viral DNA polymerase and ORF25 encoding the major capsid protein are both expressed during viral replication. ORF73 encoding the latency-associated nuclear antigen (LANA) is transcribed during viral replication and latency. ORF50 is an immediate-early gene homologous to Kaposi-sarcoma associated herpesvirus (KSHV) ORF50 and Epstein Barr virus (EBV) Rta [24]–[26]. Expression of ORF50 orthologues is thought to allow the switch from latent to productive infection. RT-PCR analysis of mononuclear cells isolated from the spleen and the popliteal lymph node of infected rabbits revealed no expression of ORF25 and ORF9. In contrast, the expression of ORF73 was detected in spleen (rabbits IR17/1 and IR20) and lymph nodes cells (rabbits IR17/2, IR20 and IR24). Finally, low levels of ORF50 expression could be detected in spleen (rabbit IR17/1) and lymph nodes cells (rabbits IR17/1 and 17/2). When reverse transcriptase was omitted from the reactions, no amplification occurred, indicating that the products of amplification did not result from contaminant viral DNA (data not shown). A β-globin control RT-PCR performed on the same samples generated the expected 249-bp product demonstrating the presence of cellular RNA. The amplification of ORF73, ORF50, ORF9 and ORF25 was controlled by using RNA extracted from AlHV-1 infected EBL (Fig. 4).

## Discussion

Based on clinical signs, macroscopical and histopathological observations, the disease induced by AlHV-1 in rabbits and in susceptible species is undistinguishable [3], [4], [27]–[30]. However, one can not claim that WD-MCF pathogenesis is strictly identical in rabbits and in natural susceptible species. In the present study, we used the rabbit model and *in vivo* BrdU incorporation to study WD-MCF pathogenesis. Our results obtained can be summarized as follows. (i) AlHV-1 infection induces CD8^+^ T cell proliferation detectable as early as 15 days post-inoculation (Fig. 2B). (ii) While the viral load in peripheral blood mononuclear cells remains below the detection level during most of the incubation period, it increases drastically few days before death (Fig. 3A). At that time, nearly all CD8^+ ^cells are positive for BrdU incorporation and at least 10% of them carry the viral genome; while CD11b^+^, IgM^+^ and CD4^+^ cells do not. (Fig.3B). (iii) RT-PCR analyses of mononuclear cells isolated from the spleen and the popliteal lymph node of infected rabbits revealed no expression of ORF25 and ORF9, low or no expression of ORF50, and high or no expression of ORF73 (Fig. 4).

Based on these results, we propose a new model for the pathogenesis of WD-MCF. According to this model, after initial replication in unidentified tissues, AlHV-1 could spread to CD8^+^ T cells or their progenitors to establish a predominantly latent infection. This non replicative infection could deregulate the infected cells leading to their proliferation and their cytotoxic behaviour [5]. The initial and restricted viral replication phase could be responsible for the development of the anti-AlHV-1 adaptive immune response. The proliferation of CD8^+^ T cells detected as early as 15 days post-inoculation (Fig. 2B, CD8 panels) while the viral charge in PBMC was still below the detection level could reflect the development of a cellular anti-AlHV-1 immune response. Further studies are required to determine the antigenic specificity of CD8^+^ T cells that proliferate during the early phase of the infection and to determine whether they contribute to eradicate cells supporting permissive and/or non permissive AlHV-1 infection.

While the viral charge was undetectable during most of the incubation period, it increased drastically few days before death (Fig. 3A). This result is consistent with earlier reports [1], [7], [8]. Interestingly, analysis of sorted cells suggested that the viral tropism amongst the sorted cell sub-populations was restricted to CD8^+^ T cells (Fig. 3B). This result is consistent with an earlier report on OvHV-2 demonstrating that most infected cells co-expressed CD3 and CD8 in vascular lesions of infected bison and cow [31]. Our analyses also revealed that most CD8^+^ T cells were supporting a non replicative infection at the time of death and consisted nearly exclusively of cells that were positive for BrdU incorporation (Fig. 2B, CD8 panels). Even if the data of the present study relied on relative measures, they demonstrate that the ratio of infected versus non infected CD8^+^ T cells increased drastically when WD-MCF developed. This observation could be explained by two phenomena that are not mutually exclusive. Firstly, it is possible that infected CD8^+^ T cells contribute to the deletion of non infected cells through fratricide cytotoxicity as described for other viral infection models [32]–[34]. Secondly, it is possible that infected CD8^+^ T cells have a division rate higher than non infected CD8^+^ T cells. Further experiments are required to test these hypotheses.

The data of the present study suggest that division of cells supporting a non replicative infection rather than viral replication should be responsible for the increase of viral load associated with the disease. According to this model, AlHV-1 genome should be transmitted to both daughter cells upon cell division. LANA orthologues have been shown to play an essential role in the persistence of viral episome through cell division during latency [35]–[38]. The data presented in Figure 4 demonstrate that ORF73 encoding AlHV-1 LANA is expressed in infected organs while ORF25 and ORF9 are not. Interestingly, ORF50 was detected in 2 rabbits. It has been demonstrated for other gammaherpesviruses that ORF50 transcription is actively repressed by ORF73 during latency [39], [40]. However, during Kaposi-sarcoma associated herpesvirus (HHV-8) infection, which is essentially latent, high expression of ORF73 along with low expression of very few immediate-early lytic genes including ORF50 can be detected [41], [42]. In the case of HHV-8, the infection is believed to be abortive *in vitro* with the viral infection going to latency directly after infection. Although ORF73 can inhibit ORF50 expression and avoid viral reactivation, ORF50 is still expressed at low levels while no viral particles are produced [43], [44]. Though it is tempting to suggest that such a phenomenon occurs during WD-MCF in rabbits, further experiments are required to test this hypothesis. Previous reports showed that the OvHV-2 genome has a circular conformation in sheep PBMC, while infected bovine or rabbit LGL contained a mixture of circular and linear viral genome [45], [46]. It will be interesting in the future to determine the conformation of AlHV-1 genome in infected CD8^+^ cells and to unravel the roles of AlHV-1 ORF73 in the pathogenesis of WD-MCF. The recent cloning of AlHV-1 as an infectious BAC clone will be a valuable tool to produce the recombinants required to address this question [12]. Similarly, this BAC will be useful to identify the genes that are responsible for the deregulation of the infected cells. With that goal in mind, we recently constructed a deleted virus for the A5 gene encoding a viral G-protein coupled receptor homolog, whose expression was shown not to be essential for the induction of WD-MCF in rabbits [15].

The pathogenesis of MCF has been the subject of considerable debate. To accommodate the detection of very few infected cells in lesions, scenarios involving indirect mechanisms such as autoimmunity or deregulation of non infected lymphocytes were proposed [1], [4], [47]. Although the mechanisms of CD8^+^ T cell recruitment and tissue damage are still unknown, the present study demonstrates that the development of WD-MCF goes with the expansion of CD8^+^ T cells supporting a latent infection. At time of death the observation that at least 10% of CD8^+^ T cells were infected suggest that these cells replicate intensively and/or lead to the deletion of non infected cells. Further studies are under progress to address these questions.
